# Development of a simplified prediction model for diagnosing progressive central precocious puberty using clinical and pelvic ultrasound parameters

**DOI:** 10.1371/journal.pone.0323549

**Published:** 2025-05-09

**Authors:** Kyungchul Song, Eunju Lee, Hye Sun Lee, Hana Lee, Hyun Wook Chae, Hyun Joo Shin

**Affiliations:** 1 Department of Pediatrics, Gangnam Severance Hospital, Yonsei University College of Medicine, Seoul, Republic of Korea; 2 Biostatistics Collaboration Unit, Yonsei University College of Medicine, Seoul, Republic of Korea; 3 Department of Radiology, Research Institute of Radiological Science and Center for Clinical Imaging Data Science, Yongin Severance Hospital, Yonsei University College of Medicine, Yongin-si, Republic of Korea; Ladoke Akintola University of Technology, NIGERIA

## Abstract

This study aimed to explore the predictive value of clinical and pelvic ultrasound parameters for diagnosing central precocious puberty (CPP) and to establish a clinically useful simplified prediction model to differentiate progressive CPP (P-CP) from nonprogressive precocious puberty (N-PP). Girls aged <9 years with secondary sexual development who underwent a gonadotropin-releasing hormone stimulation test and pelvic ultrasound between September 2020 and November 2023 were retrospectively included and divided into the P-CP and N-PP groups. Logistic regression analysis was used to determine the significant parameters and develop prediction models. The diagnostic performance of the models was compared using the area under the receiver operating characteristic curve (AUC) analysis and the Delong method. The continuous net reclassification improvement (cNRI) and absolute integrated discrimination improvement (IDI) were used to determine the additive effects of ultrasound parameters. A nomogram scoring system was constructed based on a simplified model to predict the probability of developing P-CP. A total of 109 girls were included, with 64 (58.7%) in the P-CP group. Age, bone age, height, height minus midparental height, basal luteinizing hormone (LH), follicle-stimulating hormone, estradiol, insulin-like growth factor-I, Tanner stage, and cervical and fundus width were significant parameters for the diagnosis of P-CP. The models with ultrasound parameters yielded significantly higher cNRI and IDI values than the models without ultrasound parameters. The simplified model was composed of basal LH, estradiol, and fundus width that showed an AUC value of 0.93 (95% confidence interval: 0.88–0.98) with a cutoff value of 16. In conclusion, adding pelvic ultrasound parameters to traditional clinical results has an additive effect on P-CP screening. A simplified predictive model is effective for CPP screening in real-world clinics. These findings highlight the potential of the prediction model to overcome the limitations of the classical diagnostic approach for CPP in children.

## Introduction

Precocious puberty, defined as the onset of secondary sexual characteristics before 8 years in girls and 9 years in boys, is associated with early menarche, a decrease in final adult height, and psychosocial problems [1,2]. Precocious puberty is associated with medical problems such as breast cancer, obesity, and cardiovascular disease [1]. Among the etiologies of precocious puberty, central precocious puberty (CPP) is the most common and is characterized by early pubertal development induced by the premature activation of the hypothalamic-pituitary-gonadal axis [2]. The prevalence of CPP has increased in the United States, as well as in Korea, from 0.1% in 2008 to 2.5% in 2020 [3,4].

It is important to differentiate between progressive CPP (P-CP) and nonprogressive precocious puberty (N-PP) to determine the appropriate treatment for CPP [1,2]. Treatment with gonadotropin-releasing hormone (GnRH) analogs is necessary for girls with P-CP, as it will progress without proper intervention [1,5,6]. In contrast, N-PP, which includes a spectrum of conditions such as premature thelarche, exaggerated thelarche, and slowly progressive CPP, generally does not require treatment, as puberty progresses slowly and naturally, thereby avoiding early menarche [1,7]. The GnRH stimulation test is considered the most important test for differentiating P-CP and N-PP; however, it is very burdensome to children because of the multiple sampling and long examination times [2,8]. Thus, various parameters associated with precocious puberty, including growth velocity, bone age, basal luteinizing hormone (LH), and follicle-stimulating hormone (FSH) levels, are required to assess CPP [2,9]. However, relying solely on these clinical parameters to differentiate between P-CP and N-PP has limitations in terms of sensitivity and specificity [2,6]. Moreover, hormone tests, including those measuring LH and FSH, which require blood sampling, remain burdensome for children.

To address these limitations of clinical parameters in diagnosing CPP, pelvic ultrasound (US) has been proposed as an additional diagnostic tool [2]. For girls, US can be used for several purposes, such as screening for underlying ovarian tumors, evaluating ovarian or uterine maturation, and excluding adrenal pathology [10]. The morphology and size of the uterus and ovaries can be used as important indicators of advanced maturation according to age. Uterine size and morphology are more accurate than ovarian size in assessing estrogen stimulation status [10,11]. However, pelvic US is still considered a supplementary tool in the diagnosis of CPP because of the wide variation and overlaps in cutoff values, especially for the ovaries, and the diversity of measurement methods to decide which organ to include and which value should be measured for each organ during the US examination [11–13]. Because US is an operator-dependent and time-consuming examination, determining the most indicative parameters for the uterus and ovaries is essential for evaluating pediatric patients. To the best of our knowledge, no study has demonstrated a clinically effective and simplified predictive model, including pelvic US, to assess CPP, and has identified which metric, measuring the uterus or ovaries, contributes most accurately to a predictive model.

This study aimed to explore the predictive value of clinical and pelvic US parameters for diagnosing CPP and to establish a clinically useful simplified prediction model to differentiate P-CP from N-PP.

## Materials and methods

### Patients

The Institutional Review Board of our institution approved this retrospective study, and the need for informed consent was waived (Approval no. 9-2023-0232). This study was conducted in accordance with the Strengthening the Reporting of Observational Studies in Epidemiology guidelines and regulations. Electronic records of the participants were retrieved from the clinical data repository system at our center on January 17, 2024. The authors did not have access to any information that could identify individual participants. Girls aged <9 years who visited our hospital with the chief complaint of secondary sexual development between September 2020 and November 2023 were included. We selected patients who presented with breast enlargement before the age of 8 years and underwent both GnRH stimulation tests and pelvic US within 3 months of the GnRH stimulation test. Participants with other organic causes of early pubertal development, such as peripheral precocious puberty, brain tumors, a history of radiation therapy, endocrinological diseases (e.g., congenital adrenal hyperplasia), or obstetrical complications, were excluded.

P-CP was defined as children who have all of the following [6–8]: 1) Tanner stage ≥2; 2) advanced bone age of at least 1 year; 3) peak LH level ≥5 IU/L on GnRH stimulation test; and 4) peak LH/FSH ratio ≥0.6 on GnRH stimulation test. Girls who did not meet the P-CP criteria were diagnosed with N-PP.

### Clinical parameters

Height was measured to an accuracy of 0.1 cm, and body weight was recorded using an electronic scale with a precision of 0.01 kg. Body mass index (BMI) was calculated by dividing weight in kilograms by the square of the height in meters (kg/m^2^). Height, weight, and BMI were reported as standard deviation scores based on the 2017 Korean National Growth Charts [14]. Participants were stratified into three categories based on their BMI: normal (<85th percentile), overweight (85–95th percentile), or obese (≥95th percentile). Midparental height (MPH) was calculated as follows: (father’s height + mother’s height)/2 (+ 6.5 cm for boys) and (−6.5 cm for girls).

Blood samples were collected from the antecubital vein, processed immediately, and refrigerated. LH, FSH, estradiol, and prolactin levels were determined using an electrochemiluminescence immunoassay on Cobas® e801 immunoassay system, which were standardized against the second International Standard 80/552, second International Reference Preparation (IRP) World Health Organization (WHO) reference standard 78/549, CRM 6004 vis ID-GC/MS, and third IRP WHO Reference Standard 84/500, respectively (Roche Diagnostics GmbH). In the GnRH stimulation test, serum levels of LH and FSH were measured before and at 30, 60, 90, and 120 min after the injection of 100 µg of GnRH [15].

Levels of insulin-like growth factor (IGF)-I and IGF-binding protein (IGFBP)-3 were measured using an electrochemiluminescence immunoassay with the Cobas® e801 system (Roche Diagnostics GmbH). Serum concentrations of IGF-I were assessed using the Elecsys IGF-I reagent, which was calibrated against the WHO 02/254 internal standard, whereas IGFBP-3 levels were measured using the Elecsys IGFBP-3 reagent, standardized against the IDS iSYS® IGFBP-3. Bone age was assessed by an experienced pediatric endocrinologist using left-hand radiographs, according to the Greulich and Pyle-based method [16,17].

### Pelvic US evaluation

**Fig 1:**
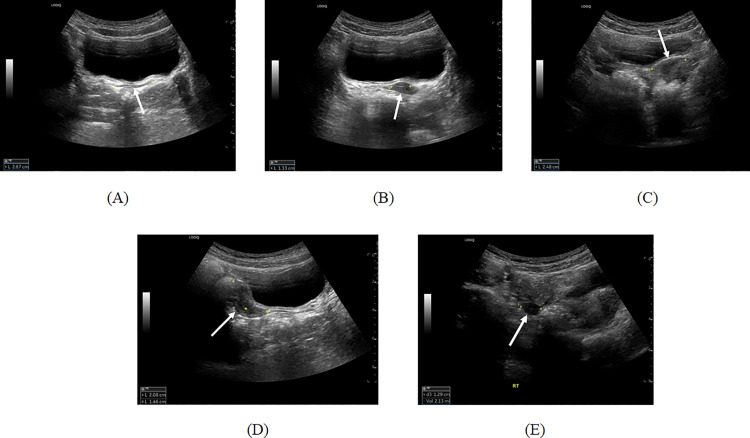
Pelvic US example of an 8-year-old girl with P-CP. US demonstrated **(a)** a vaginal width of 2.7 cm, **(b)** a cervix width of 1.3 cm, **(c)** a fundus width of 2.5 cm, **(d)** a uterine length of 3.5 cm, and **(e)** a right ovarian volume of 2.1 cc (arrows). The score for Model C was 19.4 (cutoff value > 16 for diagnosing P-CP) with a basal LH of 4.2 IU/L and estradiol of 60.8 pg/mL. US, ultrasound; P-CP, progressive central precocious puberty; LH, luteinizing hormone.

Pelvic US was performed by a board-certified pediatric radiologist with 14 years of experience, using either an Aplio i800 (Canon Medical Systems) or a LOGIQ E10 (GE Healthcare). A convex transducer of C1-8 MHz was used for the Aplio i800, and C2-9 MHz was used for the LOGIQ E10. Patients were advised to refrain from urinating for over 4 h to facilitate penetration of the US beam into the uterus and ovaries via a distended urinary bladder using the transabdominal approach. The vaginal width (cm), uterine cervix, and fundal width (cm) were evaluated in the transverse scan, and the uterine length (cm) was evaluated while rotating the transducer along the maximum diameter of the uterus (Fig 1). The uterine fundus/cervix ratio was calculated to reflect proportional changes according to age. Both ovarian sizes were measured by determining the maximum width, length, and height of the visible portion of both ovaries, and volumes (mL) were automatically calculated using US machines by multiplying with 0.523.

### Statistical analysis

All continuous variables are presented as mean ± standard deviation, whereas categorical variables are presented as numbers (percentages). After the normality test, continuous variables were compared using an independent t-test, whereas categorical variables were compared using the chi-square test or Fisher’s exact test. Univariate logistic regression analysis was performed to determine the parameters associated with P-CP, and models were constructed using significant parameters. Multivariate logistic regression analyses were performed for models that considered multicollinearity. To specifically address multicollinearity, we calculated the variance inflation factors (VIFs) for all variables included in the multivariate logistic regression models. The area under the receiver operating characteristic curve (AUC) was computed to assess the diagnostic performance of the P-CP models. The DeLong method was used for pairwise comparisons among models to test the statistical significance of differences in AUC values. However, since AUC can sometimes be insensitive to performance improvements, particularly in models with incremental changes, we also employed continuous net reclassification improvement (cNRI) and absolute integrated discrimination improvement (IDI). These metrics were calculated to more precisely assess the performance differences between models with and without US parameters. The cNRI and IDI provide additional insight into how much the inclusion of US parameters improves prediction accuracy and patient classification, offering a more nuanced understanding of model enhancement beyond what is observed with AUC alone. To present the most effective and simplified predictive model, a scoring system with a nomogram was utilized to predict the probability of P-CP using the results of the multivariable logistic regression analysis. The Youden index was used to calculate the optimal cutoff value for the total points of the scoring system. The calibration plot represented the agreement between the observed and predicted probabilities of the clinical outcomes. Data were analyzed using SAS (version 9.4; SAS Inc.) and R (version 4.3.2; The R Foundation for Statistical Computing; http://www.R-project.org). Statistical significance was set at *P* < 0.05.

## Results

### Baseline characteristics

During the study period, among the 1,085 patients who visited our hospital complaining of breast enlargement before 8 years of age, 109 girls underwent both GnRH stimulation test and pelvic US. None of the girls were excluded because of other organic causes or endocrinological diseases, including peripheral precocious puberty, as reasons for early pubertal development. Table 1 shows the baseline characteristics of the participants. Among the participants, 64 (58.7%) had P-CP and 45 had N-PP. Age, bone age, height, height - MPH, basal LH, basal FSH, peak LH, peak LH/FSH, estradiol, IGF-I, and Tanner stage were higher in participants with P-CP than in those with N-PP. Among the pelvic US parameters, mean cervix width (1.41 ± 0.36 cm vs. 1.23 ± 0.21 cm, *P* = 0.002) and fundal width (2.39 ± 0.7 cm vs. 1.95 ± 0.49 cm, *P* < 0.001) were significantly higher in the P-CP group than in the N-PP group.

**Table 1:** Baseline characteristics of the participants.

	Total (n = 109)	P-CP (n = 64)	N-PP (n = 45)	*p*
Age, year	8.42 ± 0.78	8.61 ± 0.46	8.15 ± 1.03	0.007
Bone age, year	10.31 ± 0.82	10.54 ± 0.58	9.97 ± 0.98	0.001
BA-CA, year	1.89 ± 0.66	1.94 ± 0.65	1.83 ± 0.68	0.417
Height, cm	133.76 ± 7.55	135.75 ± 7.03	130.91 ± 7.42	0.001
Height SDS	0.74 ± 1.07	0.88 ± 1.18	0.56 ± 0.86	0.104
Weight, kg	33.06 ± 7.40	34.17 ± 7.97	31.49 ± 6.24	0.062
Weight SDS	0.74 ± 0.98	0.77 ± 1.01	0.69 ± 0.95	0.676
BMI, kg/m^2^	18.35 ± 2.65	18.37 ± 2.41	18.32 ± 2.98	0.934
BMI SDS	0.55 ± 1.13	0.53 ± 0.96	0.57 ± 1.34	0.868
BMI percentile, n (%)				0.100
Normal	75 (68.81)	49 (76.56)	26 (57.78)	
Overweight	12 (11.01)	6 (9.38)	6 (13.33)	
Obesity	22 (20.18)	9 (14.06)	13 (28.89)	
MPH, cm	161.37 ± 3.48	161.01 ± 3.40	161.89 ± 3.57	0.193
MPH SDS	0.05 ± 0.70	–0.02 ± 0.68	0.16 ± 0.72	0.194
Height–MPH	0.69 ± 1.10	0.90 ± 1.20	0.40 ± 0.87	0.014
Basal LH, IU/L	1.24 ± 1.56	1.85 ± 1.79	0.36 ± 0.22	<0.001
Basal FSH, IU/L	3.57 ± 1.93	4.46 ± 1.75	2.30 ± 1.38	<0.001
Peak LH, IU/L	17.26 ± 19.51	25.68 ± 21.78	5.28 ± 2.45	<0.001
Peak FSH, IU/L	14.52 ± 4.76	14.04 ± 4.62	15.19 ± 4.94	0.217
Peak LH/FSH	1.13 ± 1.01	1.68 ± 0.99	0.35 ± 0.14	<0.001
Estradiol, pg/mL	17.03 ± 15.64	23.69 ± 17.10	7.72 ± 5.55	<0.001
Prolactin, ng/mL	17.14 ± 10.36	17.51 ± 10.00	16.63 ± 10.93	0.665
IGF-Ⅰ, ng/mL	261.06 ± 76.57	274.03 ± 80.39	218.92 ± 42.32	0.004
IGF-BP3, mcg/mL	5014.79 ± 842.44	5075.19 ± 935.78	4833.58 ± 445.37	0.239
IUP, week	38.84 ± 1.92	38.91 ± 2.22	38.76 ± 1.40	0.665
Birth weight, kg	3.09 ± 0.42	3.08 ± 0.44	3.09 ± 0.41	0.917
Yearly growth rate, year	6.47 ± 2.67	6.84 ± 2.82	5.96 ± 2.39	0.093
Tanner stage (right breast)	2.67 ± 0.64	2.86 ± 0.64	2.40 ± 0.54	<0.001
Tanner stage (left breast)	2.65 ± 0.66	2.84 ± 0.65	2.38 ± 0.58	<0.001
Vaginal width, cm	2.34 ± 0.51	2.41 ± 0.54	2.25 ± 0.44	0.091
Cervix width, cm	1.33 ± 0.32	1.41 ± 0.36	1.23 ± 0.21	0.002
Fundus width, cm	2.20 ± 0.65	2.39 ± 0.70	1.95 ± 0.49	<0.001
Uterine length, cm	3.83 ± 0.81	3.92 ± 0.97	3.70 ± 0.48	0.127
Right ovary volume, ml	2.69 ± 1.80	2.65 ± 1.98	2.74 ± 1.58	0.813
Left ovary volume, ml	2.61 ± 1.53	2.56 ± 1.49	2.67 ± 1.59	0.718
Fundus/Cervix ratio	1.69 ± 0.46	1.74 ± 0.48	1.63 ± 0.42	0.253

P-CP, progressive central precocious puberty; N-PP, nonprogressive precocious puberty; BA–CA, bone age–chronological age; SDS, standard deviation score; BMI, body mass index; MPH, mid-parental height; LH, luteinizing hormone; FSH, follicle-stimulating hormone; IGF-Ⅰ, insulin-like growth factor-Ⅰ; IGF-BP3, insulin-like growth factor-binding protein 3; IUP, intrauterine period.

### Significant clinical and pelvic US parameters for diagnosing P-CP

When we assessed the significant parameters for diagnosing P-CP using univariate logistic regression analysis, age, bone age, height, height - MPH, basal LH (per 0.1), basal FSH, estradiol, IGF-I, and Tanner stage of the right and left breast were significant factors among the clinical variables (Table 2). Among the pelvic US parameters, only the cervix width (odds ratio [OR]: 8.89, 95% confidence interval [CI]: 1.91–41.31) and fundal width (OR: 3.78, 95% CI: 1.71–8.36) were significant factors.

**Table 2:** Univariable logistic regression analyses for P-CP.

	OR (95% CI)	*p*
Age	2.31 (1.28–4.19)	0.006
Bone age	2.83 (1.49–5.36)	0.001
BA–CA	1.28 (0.71–2.32)	0.414
Height	1.12 (1.05–1.21)	0.002
Height SDS	1.36 (0.92–2.01)	0.127
Weight	1.07 (1.00–1.14)	0.068
Weight SDS	1.09 (0.74–1.61)	0.673
BMI	1.01 (0.87–1.16)	0.933
BMI SDS	0.97 (0.69–1.36)	0.859
BMI percentile		
Normal	ref	
Overweight	0.53 (0.16–1.81)	0.312
Obesity	0.37 (0.14–0.97)	0.044
MPH	0.93 (0.83–1.04)	0.194
MPH SDS	0.69 (0.40–1.21)	0.194
Height–MPH	1.60 (1.07–2.41)	0.023
Basa LH (per 0.1)	1.48 (1.21–1.81)	<0.001
Basal FSH	2.78 (1.82–4.25)	<0.001
Estradiol	1.20 (1.11–1.30)	<0.001
Prolactin	1.01 (0.97–1.05)	0.662
IGF-Ⅰ	1.01 (1.00–1.03)	0.043
IGF-BP3	1.00 (1.00–1.00)	0.39
IUP	1.04 (0.85–1.27)	0.685
Birth weight	0.95 (0.38–2.36)	0.916
Yearly growth rate	1.14 (0.98–1.32)	0.095
Tanner stage (right breast)	3.65 (1.79–7.45)	<0.001
Tanner stage (left breast)	3.44 (1.72–6.90)	<0.001
Vaginal width	1.99 (0.89–4.44)	0.094
Cervix width	8.89 (1.91–41.31)	0.005
Fundus width	3.78 (1.71–8.36)	0.001
Uterine length	1.49 (0.83–2.65)	0.181
Right ovary volume	0.97 (0.78–1.21)	0.811
Left ovary volume	0.95 (0.73–1.24)	0.715
Fundus/Cervix ratio	1.70 (0.68–4.24)	0.253

P-CP, progressive central precocious puberty; BA-CA, bone age-chronological age; SDS, standard deviation score; BMI, body mass index; MPH, mid-parental height; LH, luteinizing hormone; FSH, follicle-stimulating hormone; IGF-I, insulin-like growth factor-Ⅰ; IGF-BP3, insulin-like growth factor-binding protein 3; IUP, intrauterine period.

Considering multicollinearity, multivariable logistic regression analyses were performed with significant variables after excluding height and Tanner stage of the left breast (Table 3). Considering multicollinearity and clinical significance, we composed three prognostic models: Model A, excluding height and Tanner stage of the left breast; Model B, including bone age - chronological age instead of age and bone age in consideration of clinical significance; and Model C, including basal LH, estradiol, and fundus width after stepwise selection of the statistically significant variables in the univariable analysis. None of the variables included in the models had a VIF greater than 10; therefore, no variables were excluded because of multicollinearity (S1 Table). In Models A and B, basal LH (OR: 1.44, 95% CI: 1.01–2.05 and OR: 1.44, 95% CI: 1.02–2.05, respectively) and fundus width (OR: 6.68, 95% CI: 1.37–32.52 and OR: 6.79, 95% CI: 1.41–32.73, respectively) were significant factors. In Model C, basal LH (OR: 1.45, 95% CI: 1.11–1.89), estradiol (OR: 1.11, 95% CI 1.01–1.23), and fundus width (OR: 3.80, 95%: CI 1.09–13.20) were significant factors.

**Table 3:** Multivariable logistic regression analyses for P-CP.

	Model A		Model B		Model C	
	OR (95% CI)	*p*	OR (95% CI)	*p*	OR (95% CI)	*p*
Age	2.42 (0.57–10.20)	0.229				
Bone age	0.48 (0.11–2.20)	0.346				
BA-CA			0.44 (0.11–1.71)	0.236		
Height–MPH	1.54 (0.65–3.68)	0.326	1.57 (0.66–3.74)	0.307		
BMI percentile						
Normal	ref		ref			
Overweight	0.26 (0.02–2.83)	0.269	0.26 (0.02–2.83)	0.270		
Obesity	0.40 (0.06–2.85)	0.362	0.40 (0.06–2.83)	0.359		
Basal LH (per 0.1)	1.44 (1.01–2.05)	0.044	1.44 (1.02–2.05)	0.041	1.45 (1.11–1.89)	0.006
Basal FSH	0.91 (0.41–2.05)	0.827	0.90 (0.40–2.02)	0.801		
Estradiol	1.11 (0.98–1.26)	0.111	1.11 (0.99–1.26)	0.082	1.11 (1.01–1.23)	0.025
Yearly growth rate	1.02 (0.76–1.37)	0.914	1.02 (0.75–1.37)	0.913		
Tanner stage (right breast)	3.08 (0.73–12.95)	0.125	3.16 (0.76–13.10)	0.112		
Cervix width	0.17 (0.00–5.90)	0.328	0.17 (0.00–5.77)	0.321		
Fundus width	6.68 (1.37–32.52)	0.019	6.79 (1.41–32.73)	0.017	3.80 (1.09–13.20)	0.036

P-CP, progressive central precocious puberty; OR, odds ratio; CI, confidence interval; BA-CA, bone age-chronological age; BMI, body mass index; MPH, mid-parental height; LH, luteinizing hormone; FSH, follicle-stimulating hormone; IGF-Ⅰ, insulin-like growth factor-Ⅰ; IGF-BP3, insulin-like growth factor-binding protein 3.

### Diagnostic performance of the prediction models with or without US parameters

The AUC (95% CI), sensitivity, and specificity of each model were as follows: Model A, 0.94 (0.89–0.98), 0.86, and 0.90, respectively; Model B, 0.94 (0.89–0.98), 0.88, and 0.88, respectively; and Model C, 0.93 (0.88–0.98), 0.83, and 0.93, respectively (Table 4).

**Table 4:** AUCs of the each model of prediction of P-CP.

Model	AUC (95% CI)	*p*	Sensitivity (95% CI)	Specificity (95% CI)	Accuracy (95% CI)	PPV (95% CI)	NPV (95% CI)
Model A	0.94 (0.89–0.98)	<0.001	0.86 (0.77–0.95)	0.90 (0.82–0.99)	0.88 (0.81–0.94)	0.92 (0.85–1.00)	0.83 (0.72–0.94)
Model B	0.94 (0.89–0.98)	<0.001	0.88 (0.79–0.96)	0.88 (0.78–0.98)	0.88 (0.81–0.94)	0.91 (0.83–0.99)	0.84 (0.73–0.95)
Model C	0.93 (0.88–0.98)	<0.001	0.83 (0.74–0.93)	0.93 (0.86–1.00)	0.88 (0.81–0.94)	0.94 (0.88–1.00)	0.80 (0.69–0.91)

AUC, area under the receiver operating characteristic curve; P-CP, progressive central precocious puberty; CI, confidence interval; PPV, positive predictive value, NPV, negative predictive value

In the pairwise comparison between models with and without US parameters using the Delong method (Table 5), the differences in AUCs were not statistically significant, even after excluding the US parameters. However, the addition of US parameters yielded significantly positive values of cNRI for all models (Model A, cNRI: 1.12, 95% CI: 0.79–1.45, *P* < 0.001; Model B, cNRI: 1.16, 95% CI: 0.83–1.48, *P* < 0.001; and Model C, cNRI: 0.86, 95% CI: 0.51–1.21, *P* < 0.001). The addition of US parameters also yielded significantly positive values of IDI for all models (Model A, IDI: 0.06, 95% CI: 0.01–0.11, *P* = 0.011; Model B, IDI: 0.06, 95% CI: 0.82–0.11, *P* = 0.010; and Model C, IDI: 0.05, 95% CI: 0.01–0.09, *P* = 0.007).

**Table 5:** Comparison of the predictive performance of the P-CP with and without cervix width and fundus width.

Prediction model	AUC (95% CI)	Difference of AUC (95% CI)	*p*	cNRI (95% CI)	*p*	IDI (95% CI)	*p*
Model A							
without sonographic parameters*	0.92 (0.87–0.97)			Ref		Ref	
with sonographic parameters	0.94 (0.89–0.98)	0.01 (−0.02–0.04)	0.473	1.12 (0.79–1.45)	<0.001	0.06 (0.01–0.11)	0.011
Model B							
without sonographic parameters*	0.92 (0.87–0.97)			Ref		Ref	
with sonographic parameters	0.94 (0.89–0.98)	0.01 (−0.02–0.04)	0.393	1.16 (0.83–1.48)	<0.001	0.06 (0.02–0.11)	0.010
Model C							
without sonographic parameters*	0.92 (0.86–0.97)			Ref		Ref	
with sonographic parameters	0.93 (0.88–0.98)	0.00 (−0.04–0.04)	0.866	0.86 (0.51–1.21)	<0.001	0.05 (0.01–0.09)	0.007

*Sonographic parameters included cervix width and fundus width in model A and B and fundus width in model C.

Note: Delong method was used to perform pairwise comparisons between areas under the receiver operating curves for the parameters. Values are presented as p values.

The differences in the AUCs were not statistically different among models, even after excluding the US parameters. However, the addition of US parameters yielded significantly positive values of cNRI and IDI for all models.

P-CP, progressive central precocious puberty; AUC, area under the receiver operating characteristic curve; CI, confidence interval; cNRI, continuous net reclassification improvement; IDI, absolute integrated discrimination improvement.

### Selection of the simplified prediction model

**Fig 2:**
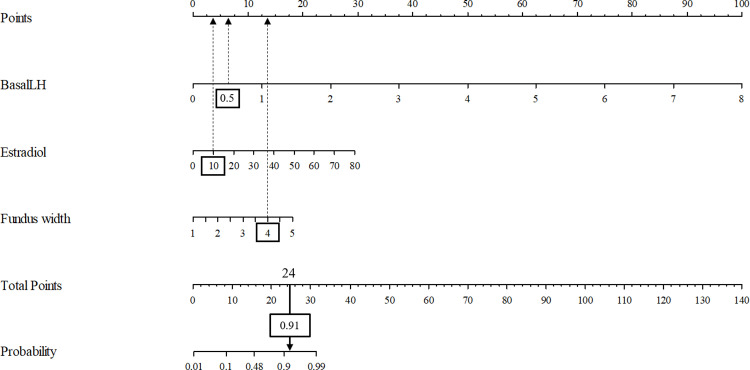
Nomogram of the Model C with pelvic US parameters. A representation of the scoring system with nomogram for prediction of P-CP using the result of multivariable logistic regression analysis. In the representation of the scoring system using nomogram, basal LH, estradiol, and fundus width of the participant were 0.5 IU/L (6 points), 10 pg/mL (4 points), and 4 cm (14 points), respectively. According to the nomogram, the probability of P-CP was 0.91 (91%) for 24 points. US, ultrasound; LH, luteinizing hormone.

A nomogram-based scoring system was developed using Model C as an effective and simplified prediction model. Fig 2 illustrates the scoring system for predicting P-CP. Model C is expressed as follows:

Probability (P-CP) = 1/ (1 + exp[-y]), where y = -6.005 + 3.689 × basal LH + 0.108 × estradiol + 1.334 × fundus width.

**Fig 3:**
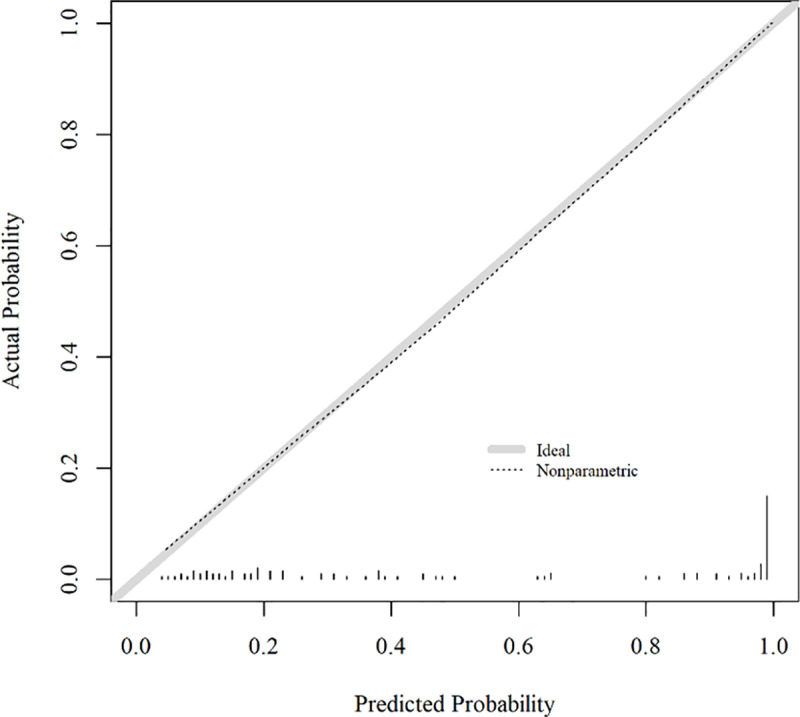
Calibration plot of the Model C with pelvic US parameters. Calibrated plots demonstrate a close approximation to the logistic calibration of each nomogram, indicating good agreement between predicted and observed outcomes when using the Model C. US, ultrasound.

In the nomogram representation of the scoring system, the basal LH, estradiol, and fundus width of the participant were 0.5 IU/L (6 points), 10 pg/mL (4 points), and 4 cm (14 points), respectively. According to the nomogram, the probability of P-CP was 0.91 (91%) for a total of 24 points. In the scoring system, the optimal cutoff point for predicting P-CP was > 16, with a corresponding probability of > 0.482. The calibration plot of the selected nomogram indicated good agreement between the predicted and observed outcomes, exhibiting a close approximation between the predicted and observed probabilities (Fig 3).

## Discussion

Our study presented an effective and simplified prediction model for the diagnosis of P-CP using clinical and pelvic US parameters. Clinical and US parameters contributed to the model’s predictive ability, with US parameters—such as cervix and fundus widths—adding incremental value. Among the multivariable models, Model C was selected as the optimal predictive model because of its clinical utility and effectiveness. It incorporates a streamlined set of parameters—basal LH, estradiol, and fundus width—identified through multivariable logistic regression with stepwise selection. Despite its simplicity, Model C achieved a high predictive accuracy, with an AUC of 0.93, comparable to more complex models. This balance of simplicity and effectiveness makes Model C particularly suitable for practical application in clinical settings. Moreover, we provided a scoring system with an optimal cutoff value; clinicians should consider more invasive tests or treatments for patients scoring above the proposed cutoff of 16, guiding decision-making in real-world settings.

The combined model, which incorporates clinical and US parameters, outperformed the model without US parameters. Since AUC can be insensitive to performance improvements, the incremental value was not clearly demonstrated in comparisons using the DeLong test. Therefore, we validated the improvement in performance using cNRI and IDI, which clearly demonstrated the incremental value added by the US parameters. To overcome the limitations of classical diagnostic methods for CPP, various investigations have been previously conducted [8,18,19]. A retrospective study demonstrated a machine learning model using clinical parameters for CPP prediction with an AUC of 0.90 in Chinese children [18]. Another retrospective study showed that a model using basal LH, estradiol, bone age, and US parameters, including uterine diameter and ovarian volume, achieved an AUC of 0.71 for P-CP [8]. Based on the effectiveness of the prediction model suggested in previous studies and the strong predictive ability demonstrated in our study, the developed nomogram scoring system of Model C may be useful for diagnosing CPP in real-world clinical practice.

Basal LH and estradiol levels were significant variables in the multivariable model after stepwise selection, which is consistent with previous study findings [8,18]. The onset of puberty begins with an increase in GnRH and sensitivity of the pituitary gland and gonads, leading to increased LH secretion and an increase in estradiol levels due to enhanced gonadal responsiveness [20]. Although basal LH and estradiol levels vary depending on the sampling time due to the body’s diurnal rhythm and pulsatile secretion patterns [21,22], they are considered useful parameters for assessing CPP [1,6,18]. International guidelines recommend basal and stimulated LH levels as the most valuable biochemical parameters for diagnosing CPP [1]. A Korean guideline suggests basal LH level as an auxiliary method for the diagnosis of CPP [6]. Carel et al. [2] suggested that the measurable estradiol level is a useful parameter for the differentiation of P-CP and N-PP. In a Chinese study, basal LH was the most important variable, followed by IGF-Ⅰ and basal FSH levels, in a prediction model for CPP [18]. In an Italian study, basal LH and estradiol levels were selected as independent predictors of rapid P-CP in a multivariable model [8].

In our study, cervix and fundus widths were identified as useful US parameters in the multivariable models. Calaterra et al. [8] suggested that longitudinal and transverse uterine diameters, along with the presence of endometrial echo, could serve as predictors of P-CP; however, these variables were not significant in our multivariable model. A Chinese study recommended using ovary and uterine volumes as parameters in a CPP prediction model [19]. In a Korean study investigating uterine volume and length, ovarian volume, and the uterine/cervix ratio, uterine volume was the only variable that showed a statistically significant difference between the CPP and control groups [23]. Another Korean study reported AUCs of 0.660 and 0.670 for uterine fundus and volume, respectively, in predicting CPP [24]. Among various measurements related to the uterus and ovaries, such as vaginal, cervix, and fundus width, uterine length, and ovarian volume, we found cervix and fundus widths to be the most useful US parameters. In addition, fundus width emerged as a more significant predictor after stepwise selection. It is widely accepted that pelvic US examinations and organ measurements additional value for diagnosing P-CP [13]. However, the organs measured and the methods used are not standardized, with different studies using varying techniques, cutoff values, and target organs. To maximize the clinical utility of pelvic US, it is essential to simplify the method by specifying which part of the organ should be measured. This is particularly important because the US examination of pelvic organs in young girls is challenging, operator-dependent, and time-consuming, especially when measuring organ dimensions. Moreover, uterine length measurements and ovarian detection may have limited reproducibility because of bladder distension and artifacts from bowel contents in the pelvic cavity. The morphological ratio of the fundus to the cervix is known to reflect hormonal status from neonates to adolescent girls [10]. These changes are clearly visible and measurable via US, making these parameters valuable indicators of hormonal status and organ maturation. Therefore, our findings regarding significant pelvic US parameters are consistent with this context. Fundus width measurement is sufficient and the most effective way to utilize the pelvic US, offering a time-efficient and easily measurable option with clinical significance for screening CPP. This approach maximizes the diagnostic utility of pelvic US in this condition.

This study has some limitations. First, as a retrospective study, it had inherent limitations. Not all patients underwent pelvic US, resulting in a smaller sample size. Additionally, since we included all patients who met the inclusion criteria during the study period, a sample size calculation was not performed in advance. Second, this study was limited to a Korean population and conducted at a single center, meaning external validation was not performed, which introduces a risk of overfitting the model and may limit the generalizability of our findings to other populations. Third, genetic studies, family history, environmental factors, and other potential confounding variables, such as the family’s socioeconomic status and diet, were not considered in this study. These factors could have influenced the observed differences between the P-CP and N-PP groups, and their absence should be acknowledged as a limitation. Fourth, while it is ideal to collect blood samples at a consistent time, particularly in the morning, to account for circadian rhythms in hormone levels, our study was conducted in a real-world clinical setting. Although we recommended morning sampling to patients and their caregivers, logistical constraints often made it challenging to perform all tests in the morning. Fifth, pelvic ultrasound and GnRH stimulation tests were conducted within a 3-month time frame, reflecting the practical constraints of collecting real-world clinic data. While this interval could introduce minor variability due to the rapid physiological changes during puberty, the impact is likely minimal as the tests were performed within a relatively short and controlled period to represent the same stage of pubertal development. Sixth, while the initial sample size was 1,085 children, only 109 children underwent pelvic ultrasound due to its high cost, selective use in challenging cases, and some patients declining the procedure due to time or financial constraints, which may introduce selection bias and limit the generalizability of our findings.

Despite the limitations, we developed a prediction model using clinical and US parameters, wherein fundus width emerged as a valuable US parameter that adds incremental value to the prediction model. Although the differences in AUC between models with and without sonographic parameters were not substantial, the clinical significance of incorporating US in our study is particularly notable given the highly invasive nature of the GnRH stimulation test typically used in pediatric assessments. This highlights the importance of integrating US, which is non-invasive and free from radiation, offering a safer alternative for the evaluation of CPP in children. From an imaging perspective, our study aimed to identify US measurements that are feasible, time-efficient, and most representative, providing key insights into streamlining the diagnostic process. The simple length measurement technique used in this study does not require specialized US skills, making it easy to implement in various clinical settings. To minimize inter-reader variability, all US evaluations were consistently performed by a single experienced radiologist. This approach enhances the potential applicability of our findings to other institutions. Despite being an initial study, its evident clinical utility adds meaningful value. Further studies involving external validation with larger samples from multi-center cohorts and diverse ethnic and demographic groups are needed to better define the role of US in enhancing the diagnostic pathway for CPP.

## Conclusion

Our study demonstrated that adding pelvic US parameters to traditional clinical results has an additive effect on P-CP screening. A simplified predictive model comprised of basal LH, estradiol, and fundus width is effective for CPP screening in real-world clinics. These findings highlight the potential of the prediction model, including US parameters, to overcome the limitations of the classical diagnostic approach for CPP in children. Further validation in diverse populations, taking into account genetic and environmental factors, is needed to confirm the broader applicability and performance of the model across different clinical settings. This will help generalize the findings of our study and clarify the utility of pelvic US parameters in assessing CPP.

## Supporting information

S1 Table Variance inflation factors of the variables for predicting P-CP. (DOCX)
