# Supplementary material for: Development of a simplified prediction model for diagnosing progressive central precocious puberty using clinical and pelvic ultrasound parameters
Source: PLoS One. 2025 May 9;20(5):e0323549. doi: 10.1371/journal.pone.0323549 (PMC12063851; doi:10.1371/journal.pone.0323549)
Supplement: S1 Table — (DOCX) [file pone.0323549.s001.docx]

**S1 Table. Variance inflation factors of the variables for predicting P-CP**

| Variance inflation factors | Model A | | Model B | | Model C | |
| --- | --- | --- | --- | --- | --- | --- |
|  | with sonographic parameters* | without sonographic parameters | with sonographic parameters | without sonographic parameters | with sonographic parameters | without sonographic parameters |
| BMI percentile |  |  |  |  |  |  |
| Normal | - | - | - | - |  |  |
| Overweight | 1.235 | 1.149 | 1.196 | 1.134 |  |  |
| Obesity | 1.266 | 1.275 | 1.254 | 1.248 |  |  |
| Age | 2.362 | 2.640 |  |  |  |  |
| Bone age | 2.654 | 2.888 |  |  |  |  |
| BA-CA |  |  | 1.630 | 1.624 |  |  |
| Height–MPH | 1.829 | 1.649 | 1.823 | 1.639 |  |  |
| Basal LH (per 0.1) | 2.322 | 2.071 | 2.317 | 2.066 | 1.527 | 1.385 |
| Basal FSH | 3.336 | 3.136 | 3.319 | 3.133 |  |  |
| Estradiol | 2.439 | 2.310 | 2.368 | 2.248 | 1.397 | 1.385 |
| Yearly growth rate | 1.115 | 1.097 | 1.115 | 1.094 |  |  |
| Tanner stage (right breast) | 1.364 | 1.267 | 1.331 | 1.244 |  |  |
| Cervix width | 1.745 |  | 1.744 |  |  |  |
| Fundus width | 1.631 |  | 1.625 |  | 1.177 |  |

*Sonographic parameters included cervix width and fundus width in model A and B and fundus width in model C.

P-CP, progressive central precocious puberty; BMI, body mass index; BA-CA, bone age-chronological age; MPH, mid-parental height; LH, luteinizing hormone; FSH, follicle-stimulating hormone.
